# Role of CGRP in Neuroimmune Interaction via NF-κB Signaling Genes in Glial Cells of Trigeminal Ganglia

**DOI:** 10.3390/ijms21176005

**Published:** 2020-08-20

**Authors:** Shaista Afroz, Rieko Arakaki, Takuma Iwasa, Arief Waskitho, Masamitsu Oshima, Yoshizo Matsuka

**Affiliations:** 1 Department of Prosthodontics and Dental Material, ZA Dental College, Aligarh Muslim University, Aligarh 202002, India; shaista_afroz@yahoo.com; 2 Department of Oral Molecular Pathology, Graduate School of Biomedical Sciences, Tokushima University, Tokushima 700-8504, Japan; arakaki.r@tokushima-u.ac.jp; 3 Department of Stomatognathic Function and Occlusal Reconstruction, Graduate School of Biomedical Sciences, Tokushima University, Tokushima 700-8504, Japan; iwasatakuma0904@outlook.jp (T.I.); arief.waskitho.85@gmail.com (A.W.); m-oshima@tokushima-u.ac.jp (M.O.)

**Keywords:** satellite glial cells, calcitonin gene related peptide (CGRP), nuclear factor kappa B (NF-κB), trigeminal ganglion, glial cells

## Abstract

Activation of the trigeminal system causes the release of various neuropeptides, cytokines, and other immune mediators. Calcitonin gene-related peptide (CGRP), which is a potent algogenic mediator, is expressed in the peripheral sensory neurons of trigeminal ganglion (TG). It affects the inflammatory responses and pain sensitivity by modulating the activity of glial cells. The primary aim of this study was to use array analysis to investigate the effect of CGRP on the glial cells of TG in regulating nuclear factor kappa B (NF-κB) signaling genes and to further check if CGRP in the TG can affect neuron-glia activation in the spinal trigeminal nucleus caudalis. The glial cells of TG were stimulated with CGRP or Minocycline (Min) + CGRP. The effect on various genes involved in NF-κB signaling pathway was analyzed compared to no treatment control condition using a PCR array analysis. CGRP, Min + CGRP or saline was directly injected inside the TG and the effect on gene expression of *Egr1*, *Myd88* and *Akt1* and protein expression of cleaved Caspase3 (cleav Casp3) in the TG, and c-Fos and glial fibrillary acidic protein (GFAP) in the spinal section containing trigeminal nucleus caudalis was analyzed. Results showed that CGRP stimulation resulted in the modulation of several genes involved in the interleukin 1 signaling pathway and some genes of the tumor necrosis factor pathway. Minocycline pre-treatment resulted in the modulation of several genes in the glial cells, including anti-inflammatory genes, and neuronal activation markers. A mild increase in cleav Casp3 expression in TG and c-Fos and GFAP in the spinal trigeminal nucleus of CGRP injected animals was observed. These data provide evidence that glial cells can participate in neuroimmune interaction due to CGRP in the TG via NF-κB signaling pathway.

## 1. Introduction

Neuroimmune interaction causes the development of sensitization of pain. Immune activation and the subsequent release of immune mediators in the peripheral nervous system (PNS) contribute to pain [1,2]. The release of cytokines and inflammatory mediators from the peripheral glial cells (satellite glial cells (SGC) or Schwann cells) or blood derived immune cells (mast cells, macrophages, and lymphocytes) in response to some trauma or noxious stimuli to the PNS, has been shown by various researchers to be responsible for causing peripheral and central sensitization, thereby contributing to the development of allodynia and hyperalgesia, two hallmarks of neuropathic pain (NP) [1,2,3,4]. Different kinds of sciatic nerve injury lead to the activation of nuclear factor kappa B (NF-κB) in dorsal root ganglion (DRG) neurons and in some Schwann cells surrounding unmyelinated fibers in a chronic constriction injury model (CCI) [5]. NF-κB can be activated by numerous endogenous or exogenous molecules, which by translocating to the nucleus can modulate the expression of proinflammatory genes, such as those for inducible nitric oxide synthase (*iNOS*), cyclo-oxygenase-2, adhesion molecules, cytokines, and chemokines [6]. NF-κB plays an essential role in the gene expression of inflammatory mediators in different cell types, including neurons and glial cells. Transgenic inhibition of glial NF-κB has been shown to inhibit hyperalgesia and allodynia in mice, and these effects were related to a reduction in cytokine/chemokine production in DRGs [7].

Activation of the trigeminal system has been shown to be associated with the release of vasoactive neuropeptides such as calcitonin gene-related peptide (CGRP) and substance P (SP). CGRP is a potent algogenic mediator, contributing to the development of peripheral and central sensitization in orofacial inflammatory pain, NP, migraine and medication overuse-related headache [8,9,10], and a mediator of neuroimmune communication [11]. It affects the inflammatory responses and pain sensitivity by modulating the activity of glial cells [8]. CGRP was demonstrated to contribute to the development of tolerance to morphine-induced analgesia partly through induction of p38 mitogen-activated protein kinases (MAPK) phosphorylation and activation of the NF-κB signaling pathway in spinal cord microglial cells [12]. Injection of CGRP stimulates the activation of spinal cord microglia in a model of temporomandibular joint disorder, as evidenced by increased OX-42 immunostaining [8]. Indeed, several findings support the role of SGC and Schwann cells in regulating responses to pain in the trigeminal ganglion (TG) and DRG [4]. In our previous research, we observed that when CGRP was injected into the TG, it resulted in thermal hyperalgesia in first 6 h, and this was accompanied by an increase in the gene expression of proinflammatory cytokine IL-1β and IL-6 and anti-inflammatory cytokine IL-1RA, as well as an increase in the expression of glial fibrillary acidic protein (GFAP), a marker of glial activation [13]. When the glial cells were exposed to CGRP overnight, it resulted in the differential expression of several cytokines [13], a finding supported by other studies as well [14,15,16].

It has been shown that NF-κB plays a crucial role in regulating the gene expression of proinflammatory substances, resulting in hyperalgesia [5,17]. Data from degenerative disc disease patients have shown that the NF-κB signaling by peptides including CGRP may be involved in the peripheral pain mechanism [18]. Former studies have shown that activation of NF-κB cascade in response to stress or injury can mediate a variety of cellular responses which can cause inflammatory or NP [19,20]. The NF-κB signaling pathway is shown to be a mediator of intervertebral disc degeneration and spinal nerve ligation-induced NP [19,20]. Different studies have shown that the therapeutic agent which targets the NF-κB pathway, for example ligand/receptors or adapter proteins or transcription factors, and others, have an anti-inflammatory, neuroprotective, and analgesic effect [21,22,23,24,25]. In a model of cerebral ischaemia, *N*-palmitoylethanolamide-oxazoline exerted its anti-inflammatory and neuroprotective effect by restoring the expression of inhibitor of kappa B-α (IκBα) and reducing the translocation of NF-κB in nucleus [21]. Reduced binding to or inhibition of toll-like receptor (Tlr) 4 has been shown to be effective in reducing mechanical allodynia and thermal hyperalgesia in animal model of NP [22,23]. This effect was due to inhibition of NF-κB signaling pathway. Myeloid differentiation factor-88 adaptor protein (Myd88) mediates activation of Tlrs or interleukin 1 receptor (IL1-R) and leads to NF-κB activation in peripheral neuropathy and has been shown to be a therapeutic target for NP [24,25]. The findings of the former studies give the insight to explore CGRP induced NF-κB-related responses in glial cells in TG. Therefore, the aim of this investigation was to elucidate the NF-κB activity on the glial cells of TG after CGRP treatment and to further check if CGRP in the TG can affect neuron-glia activation in the spinal trigeminal nucleus caudalis.

## 2. Results

### 2.1. CGRP Induces Differential Regulation of Several Genes in Glial Cells of TG Involved in NF-κB Signaling Pathway

Overnight stimulation with 1 µM CGRP lead to differential regulation of various genes involved in NF-κB signaling (Table 1). In total, 84 genes were included in the PCR array analysis. Eleven were removed from analysis because of the following reasons: this gene’s, (interferon gamma) average threshold cycle (*C*_T_ value) was not determined or greater than the defined cut-off (35), in both samples, meaning that its expression was undetected; or these gene’s (chemokine (C-C motif) ligand *(Ccl) 5*, colony stimulating factor *(Csf) 2,* interferon-alpha 1, lymphotoxin alpha, and tumor necrosis factor superfamily, member *(Tnfs) 14)* average threshold cycle is relatively high (> 30) in either the control or the test sample, and is reasonably low in the other samples (< 30) and *p* value > 0.05; or these gene’s (caspase recruitment domain family, member 11, *Csf3*, *Tnfsf10*, zeta-chain (TCR) associated protein kinase *(Zap70))* average threshold cycle is relatively high (> 30), meaning that its relative expression level is low, in both control and test samples, and the *p*-value for the fold-change is either unavailable or relatively high (*p* > 0.05). Therefore, a greater number of replicates will be required for these genes to validate the results.

After CGRP stimulation, the expression of 61 genes were upregulated (more than 1.5-fold change), three were down regulated (fold regulation less than 1) and 11 genes were unchanged (1–1.5-fold) compared to controlled condition (untreated group) (Table 1).

**Figure 1:**
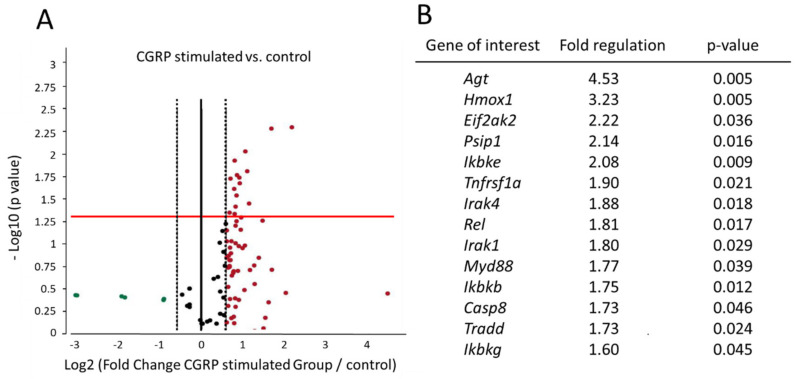
Fold regulation of various genes of NF-κB signaling pathway in the glial cells of trigeminal ganglion (TG) after overnight stimulation with 1 μM CGRP compared to no treatment control group. (**A**) Volcano plot showing RT-PCR array data to compare the normalized expression of all the genes included in the array between the CGRP stimulated group and control group (no treatment group). The red dots are the genes which were upregulated, and green dots were the genes which were down regulated. The red line shows where *p* = 0.05 with points above the line having *p* < 0.05 and points below the line having *p* > 0.05. Dots above the red line indicate genes-of-interest that display both more than 1.5-fold-changes (*x* axis) and *p* < 0.05, statistical significance (−log10 of *p* value, *y* axis). Those points having a fold-change less than 1.5 are shown in black dots. (**B**) Genes-of-interest, which were significantly upregulated after overnight stimulation with 1 micromolar CGRP compared to the no treatment control group. Fold Regulation cut off: 1.5; *p*-Value cut off: 0.05, *n* = 3.

A significant upregulation (fold regulation > 1.5, and *p* < 0.05) was noted for 14 genes which were involved in NF-κB signaling pathway (Figure 1). Tumor necrosis factor receptor superfamily, member 1a *(Tnfrsf1a)* was significantly upregulated (fold regulation = 1.90, *p* = 0.021) and the protein encoded by this gene is one of the major receptors for the tumor necrosis factor α (TNF-α). Proteins encoded by the following genes are responsible for downstream signaling of NF-κB and were upregulated: interleukin-1 receptor associated kinase (*Irak*) 1 (fold regulation = 1.80, *p* = 0.029), *Irak4* (fold regulation = 1.88, *p* = 0.018), *Myd88* (fold regulation = 1.77, *p* = 0.039) and TNFRSF1A-associated via death domain *(Tradd)* (fold regulation = 1.73, *p* = 0.025). Proteins encoded by the genes involved in cytoplasmic sequestering/releasing of NF-κB showed an upregulation: inhibitor of kappa light polypeptide gene enhancer in B-cells, kinase beta (*Ikbkb)* (fold regulation = 1.75, *p* = 0.012), inhibitor of kappa light polypeptide gene enhancer in B-cells, kinase epsilon *(Ikbke)* (fold regulation = 2.08, *p* = 0.009), inhibitor of kappa light polypeptide gene enhancer in B-cells, kinase gamma *(Ikbkg)* (fold regulation = 1.60, *p* = 0.045), and transcription factor V-rel avian reticuloendotheliosis viral oncogene homolog *(Rel)* was also upregulated (fold regulation = 1.81, *p* = 0.017). NF-κB responsive gene angiotensinogen *(Agt)* (fold regulation = 4.53, *p* = 0.005) expression was highly significant. Other genes which were significantly upregulated were kinase: eukaryotic translation initiation factor 2-alpha kinase 2 *(Eif2ak2)* (fold regulation = 2.22, *p* = 0.035), caspase 8 *(Casp8)* (fold regulation = 1.73, *p* = 0.046), heme oxygenase (decycling) 1 *(Hmox1)* (fold regulation = 3.23, *p* = 0.005), and PC4 and SFRS1 interacting protein 1 *(Psip1)* (fold regulation = 2.14, *p* = 0.016).

### 2.2. Effect of Minocycline Treatment for 30 Min Followed by CGRP Stimulation on NF-κB Pathway’s Signaling Genes

Minocycline is considered to be a putative glial inhibitor. In our previous research we noted that intraganglionic (IG) Minocycline was effectively able to cause glial inhibition, prevent the pro-nociceptive effect of IG CGRP, and decrease the gene expression of IL1-β and IL-6 [13]. So, in the present study, the effect of glial inhibition on the NF-κB signaling genes was checked by treating the glial cells rich culture with minocycline followed by overnight stimulation with CGRP (Min + CGRP group) (Table 1).

**Figure 2:**
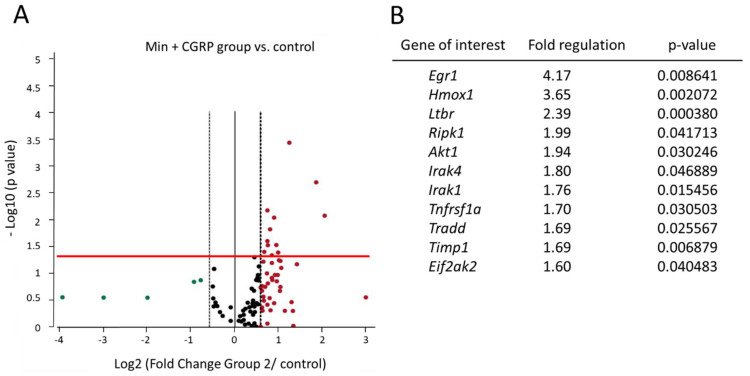
Fold regulation of various genes of NF-κB signaling pathway in the glial cells of TG after 30 min treatment with Minocycline followed by overnight stimulation with 1 μM CGRP (Min + CGRP) compared to no treatment control group. (**A**) Volcano plot showing RT-PCR array data to compare the normalized expression of all the genes included in the array between the Min + CGRP group and control group (no treatment group). The red dots are the genes which were upregulated, and green dots were the genes which were down regulated. The red line shows where *p* = 0.05 with points above the line having *p* < 0.05 and points below the line having *p* > 0.05. Dots above the red line indicate genes-of-interest that display both more than 1.5 fold-changes (*x* axis) and *p* < 0.05, statistical significance (−log10 of *p* value, *y* axis). Those points having a fold-change less than 1.5 are shown in black dots. (**B**) Genes-of-interest, which were significantly upregulated in Min + CGRP group compared to the no treatment control group. Fold Regulation cut off: 1.5; *p*-Value cut off: 0.05, *n* = 3.

NF-κB signaling genes which were differentially upregulated (fold regulation > 1.5, and *p* < 0.05) in Min + CGRP group are: V-akt murine thymoma viral oncogene homolog 1 *(Akt1)* (kinase, fold regulation = 1.94, *p* = 0.030), early growth response 1 *(Egr1)* (transcription factor, fold regulation = 4.17, *p* = 0.008), lymphotoxin beta receptor (TNFR superfamily, member 3) (*Ltbr*) (NF-κB ligand and receptor, fold regulation = 2.39, *p* < 0.001), receptor-interacting serine-threonine kinase 1 (*Ripk1*) (downstream signaling gene, fold regulation = 1.99, *p* = 0.042), and tissue inhibitor of metalloproteinases 1 (*Timp1*), (fold regulation = 1.69, *p* = 0.007). Certain genes whose expression level was significantly upregulated (fold regulation > 1.5, *p* < 0.05) in the CGRP group was also upregulated in Min + CGRP group) and were *Eif2ak2* (fold regulation = 1.60, *p* = 0.040), *Hmox1* (fold regulation = 3.65, *p* = 0.002), *Irak1* (fold regulation = 1.76, *p* = 0.015), *Irak4* (fold regulation = 1.80, *p* = 0.046), *Tnfrsf1a* (fold regulation = 1.70, *p* = 0.031), and *Tradd* (fold regulation =1.69, *p* = 0.026) (Figure 2).

A comparison between CGRP stimulated group and Min + CGRP group showed upregulation of Nuclear factor of kappa light polypeptide gene enhancer in B-cells inhibitor, alpha *(Nfkbia)* (fold regulation = 1.74, *p*-value = 0.002), *Rel* (fold regulation = 1.47, *p*-value = 0.008), *Psip1* (fold regulation = 1.47, *p*-value = 0.011), Chemokine (C-C motif) ligand 2 *(Ccl2)* (fold regulation = 2.59, *p*-value = 0.016), *Tlr3* (fold regulation = 1.48, *p*-value = 0.021), *Agt* (fold regulation = 2.7, *p*-value = 0.028), *Ibkke* fold regulation = 1.47, *p*-value = 0.032), and downregulation of *Egr1* fold regulation = −2.53, *p*-value = 0.007), Caspase recruitment domain family, member 10 *(Card 10)* (fold regulation = −1.47, *p*-value = 0.015), and *Ltbr* (fold regulation = −1.62, *p*-value = 0.042) (Table 1).

### 2.3. Effect of IG CGRP Administration and Minocycline Treatment Followed by CGRP Stimulation on the Gene Expression of Myd88, Egr1 and Akt1

**Figure 3:**
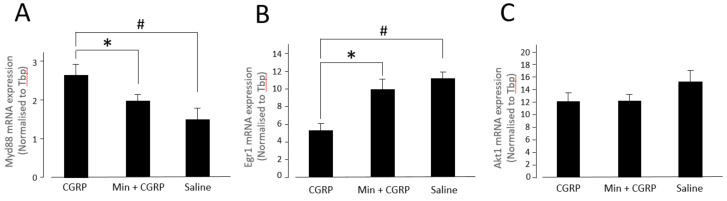
In array analysis CGRP stimulation of glial cells lead to an increased expression of *Myd88* and Minocycline treatment before CGRP stimulation lead to an increased expression of *Egr1* and *Akt1* genes. Therefore, the effect of intraganglionic (IG) CGRP and Min + CGRP on mRNA expression of *Myd88*, *Egr1* and *Akt1* in Trigeminal ganglion after 6 h (In vivo) was analyzed. (**A**) An increase in mRNA expression of *Myd88* was observed 6 h after CGRP injection compared to Saline injected control group. When Minocycline was injected 1 h before CGRP, decrease in the mRNA expression of *Myd88* was observed compared to only CGRP injected group. (**B**) A decrease in mRNA expression of *Egr1* was observed 6 h after CGRP injection compared to Saline injected control group. When Minocycline was injected 1 h before CGRP, an increase in the mRNA expression of *Egr1* was observed compared to only CGRP injected group. (**C**) No statistical difference was observed in between any group in the mRNA expression of *Akt1*. # *p* < 0.05 with one-way ANOVA followed by the Dunnett *t* test for comparison with saline injection. * *p* < 0.05 with *t* test, *n* = 5 rats were assigned to each group.

In a different experiment set up, the effect of IG CGRP, Min + CGRP and only saline injection on the expression of some of the genes of NF-κB signaling pathway in TG was checked by quantitative real-time polymerase chain reaction (qRT PCR). mRNA expression of *Myd88* (which was significantly elevated in CGRP stimulated group and is related to downstream signaling of NF-κB), *Akt1* and *Egr1* (which were significantly elevated in Min + CGRP group) were selected for qRT PCR analysis (Figure 3A–C). In our previous research we noted that the highest effect of IG injection of CGRP and Min + CGRP on gene expression and behavior changes was 6 h after drug administration. This latency is stipulated as necessary for upregulation of genes for pathogenesis of pain [26]. So, for *Myd88*, *Akt1* and *Egr1* analysis TG samples were collected 6 h after IG drug administration. There was a significant effect of injecting drugs on the expression level of *Myd88* [F (2,12) = 5.979, *p* = 0.016] and *Egr1* [F (2,12) = 9.245, *p* = 0.004] at the *p* < 0.05 level for the three injected groups (Figure 3A,B). For the mRNA expression of *Myd88* post hoc comparison using Dunnett *t* test indicated that the mean score of CGRP injected group (M = 2.678, SD = 0.560) was significantly higher than the saline injected group (M = 1.47, SD = 0.678). However, the Min + CGRP (M = 1.98, SD = 0.38) injected group did not differ significantly from only saline injected group. Independent-samples *t*-test showed significant difference between CGRP and Min + CGRP injected groups; *t* (8) = 2.313, *p* = 0.049, on the expression of *Myd88*. For the mRNA expression of *Egr1* post hoc comparison using Dunnett *t* test indicated that the mean score of CGRP injected group (M = 5.2, SD = 2.1) was significantly different from the saline injected group (M = 11.1, SD = 1.84). However, Min + CGRP (M = 9.9, SD = 2.9) injected group did not differ significantly from the only saline injected group. Independent-samples *t*-test showed significant difference between CGRP and Min + CGRP injected groups; *t* (8) = −2.977, *p* = 0.018, on the expression of *Egr1*. There was no significant effect of injecting drugs on the expression level of *Akt1* [F (2,12) = 2.139, *p* = 0.161] at the *p* < 0.05 level for the three injected groups (Figure 3C).

### 2.4. Effect of CGRP Stimulation on Cleaved Caspase-3 (Cleav Casp3) Protein Expression of TG

**Figure 4:**
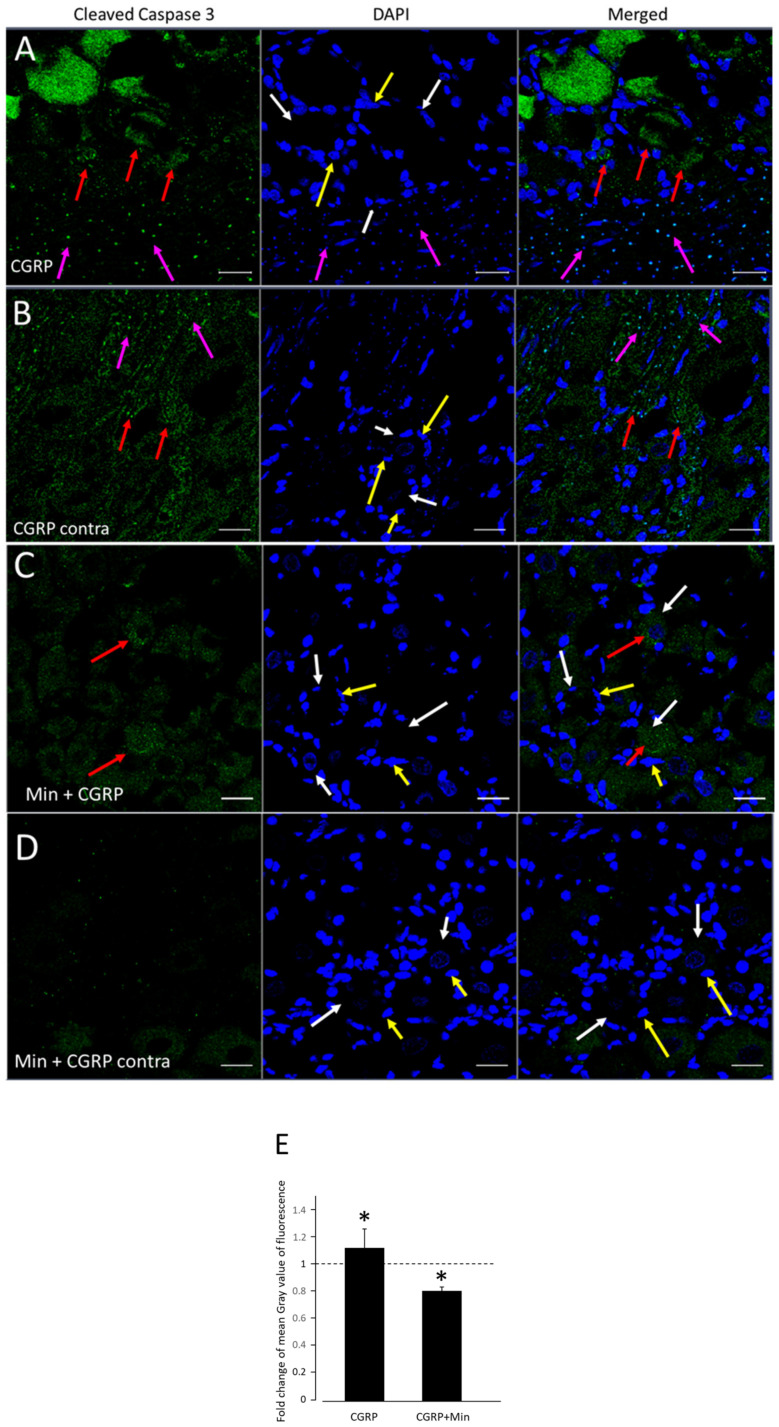
Following the NF-κB signaling pathway gene analyses, where fold regulation of Casp8 was found to be increased after CGRP stimulation, change in expression of cleaved Caspase-3 (cleav Casp3) in sections from TG was observed in the animals after IG administration of CGRP and Min + CGRP compared to non-injected ganglion. Confocal images of immunofluorescent staining of TG sections with cleav Casp3 (green immunofluorescence), and 4′,6-Diamidino-2-phenylindole dihydrochloride (DAPI, blue) 6 h after IG CGRP and Min + CGRP administration and non-injected contralateral TGs were observed and staining intensity was compared. Neuronal profiles were identified as the areas (white arrows) surrounded by satellite glial cells (SGC) (yellow arrows). (**A**) There was an increased in immunoreactivity of cleav Casp3 in CGRP injected sections. The cleav Casp3 immunoreactivity was observed in the neuronal and SGCs cytoplasm (red arrow), whereas as non-neuronal cells showed a colocalization with DAPI (purple arrow), (**B**) similar distribution pattern was observed in the non-injected contralateral side, but the overall staining intensity was low, (**C**) and (**D**) Min + CGRP injected group and non-injected contralateral side showed marked decrease in staining intensity in TG. (**E**) The average fold change ± SEM of cleav Casp3 staining intensity from control values. A mild but significant increase in fold change in mean grey value of cleav Casp3 was found 6 h after CGRP injection on the injected side compared to the non-injected contralateral side. When Minocycline was injected one hour before CGRP, there was a significant decrease in fold change in mean grey value of cleav Casp-3 on the injected side compared to non-injected side. * *p* < 0.05 with *t* test for two dependent means, *n* = 5. Independent-samples *t*-test showed no significant difference between CGRP and Min + CGRP injected groups. Scale bar 20 μm.

The caspase responsible for the initiation of apoptosis, caspase-8 (Casp8) (fold regulation = 1.73, *p* = 0.046), was found to be up-regulated in PCR array analysis of glial cells post-CGRP stimulation. Therefore, to check the effect of CGRP injection on the apoptosis of TG cells, the expression of cleav Casp3 (active form), which is the executioner of apoptosis and exists within the cytosol as inactive dimers as Casp3, was checked on frozen sections of TG using immunohistochemistry (Figure 4A). A mild but significant increase in fold change in mean grey value of cleav Casp3 was found 6 h after CGRP injection on the injected side (M = 1.119; SD = 0.323) compared to the non-injected contralateral side (M = 1; SD = 0.277); t (4) = −3.131, *p* = 0.0351) as calculated using *t* test for two dependent means at the *p* < 0.05 level (Figure 4B). When Minocycline was injected one hour before CGRP, there was a significant decrease in fold change in mean grey value of cleav Casp3 on the injected side (M = 0.802911; SD = 0.0650) compared to non-injected side (M = 1.00; SD = 0.0729); (t (4) = 3.461, *p* = 0.0258) using the *t* test for two dependent means at the *p* < 0.05 level. Independent-samples *t*-test showed no significant difference between CGRP and Min + CGRP injected groups (t (8) = 2.1453, *p* = 0.0642) on the expression of cleav Casp3 (Figure 4B).

### 2.5. Effect of IG CGRP Administration on the Spinal Trigeminal Nucleus Caudalis Glial and Neuronal Activation—A Qualitative Assessment

**Figure 5:**
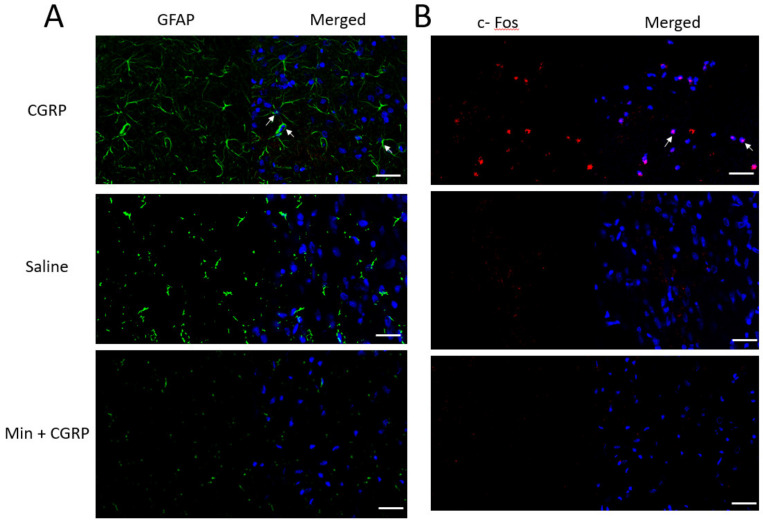
Following the administration of CGRP, Min + CGRP and saline in the TG the change in activity of neurons and astroglial cells in spinal trigeminal nucleus cells was qualitatively observed. Change in expression of glial fibrillary acidic protein (GFAP) in astrocytes, a marker of glial activation and neuronal c-Fos, an early immediate transcription factor and marker of second order sensory neurons in spinal trigeminal nucleus sections of spinal cord obtained from animals with IG administration of CGRP, saline and Min + CGRP was observed. (**A**) Immunohistochemical staining of spinal trigeminal nucleus caudalis section with GFAP (green) and DAPI (blue). An increase in expression of GFAP (green) in astrocytes (white arrow) in spinal trigeminal nucleus was observed 6 h after IG CGRP administration compared to IG administration of saline or Min + CGRP, (**B**) An increase in expression of neuronal C-Fos (red) and DAPI (blue) was observed 6 h after IG CGRP administration compared to only saline or Min + CGRP group (white arrow). *n* = 3. Scale bar 20 μm.

c-Fos is a member of early immediate transcription factor, and increased in its expression is related to the activation of second order sensory neurons within the spinal medullary horn. An increased in expression of c-Fos positive neurons was observed when CGRP was injected in TG, compared to only saline and Min + CGRP injected neurons (Figure 5B). GFAP is an intermediate cytoskeleton filament protein and is a biomarker of astrocyte activation. An increase in expression of the cytoskeletal protein GFAP was observed in the only CGRP injected group compared to only saline and Min + CGRP injected group, thus indicating an enhanced level of activity of astrocytes in the spinal trigeminal nucleus caudalis (Figure 5A).

## 3. Discussion

In this study, the genes which were included in the PCR array analysis were related to the various steps involved in NF-κB signaling pathway, namely various ligands and receptors regulating NF-κB signaling, genes related to the downstream signaling of NF-κB, cytoplasmic sequestering/releasing of NF-κB, transcription factors, NF-κB responsive genes, e.g., immune response and apoptosis, and other NF-κB signaling genes, e.g., kinases and transcription factors. In our previous research, we found that CGRP induced differential regulation of various cytokines from glial rich culture of TG [13]. The mRNA expression of cytokines IL1-β, IL-1RA, and IL-6 were also upregulated in TG, post CGRP administration in the TG [13]. Many of the proinflammatory effects of IL-1 are exerted at the level of transcription regulation which is mediated by NF-κB [27]. Therefore, in the present study, the effect of CGRP on the expression of NF-κB signaling genes was evaluated on the glial rich culture of TG. Overnight stimulation of glial rich culture resulted in the differential expression of several genes involved in NF-κB signaling compared to the control condition. *Tnfrsf1a* the protein encoded by this gene is one of the major receptors for the TNF-α, the genes involved in downstream signaling leading to NF-κB activation, *Myd88*, *Irak1*, *Irak4*, *Tradd*, *Ikbkb*, *Ikbke*, *Ikbkg*, and the transcription factor *Rel* were significantly upregulated after CGRP stimulation (Figure 1B). Proinflammatory signals, bind to their corresponding receptors, leading to a recruitment of receptor-associated proteins, such as *Myd88* and *Irak* for IL-1R/Tlr, and *Tradd* and *Rip1* for TNF receptor [28]. This may lead to phosphorylation and activation of *Ikk* (*Ikbkb*, *Ikbke*, *Ikbkg*) complexes that are responsible for the phosphorylation and/or degradation of the IkB protein thereby release of NF-κB [20]. Thus, the activation of these genes triggers the pathway leading to activation of NF-κB, and the production of proinflammatory cytokines. In TG, CGRP receptors are found on large diameter neurons and glial cells including SGCs and Schwann cells [29,30,31], whereas SGCs, from human TG, are reported to have a broad expression of Tlr including Tlr1–4 and 9 and secrete the proinflammatory cytokines IL-6 and TNF-α upon specific Tlr stimulation [32]. CGRP receptors activation has been reported to increase in activity of MAPK [33]. It is also reported that the activation of CGRP receptors on trigeminal glial cells by CGRP stimulation leads to an increased expression of the active P-c Jun amino-terminal kinase (JNK) and P-p38, and an augmented activity of the MAPK-responsive genes *Elk1*, *Atf2*, and C/EBP homologous protein (CHOP), which function as transcription factors to regulate inflammatory gene expression, in trigeminal ganglion glial cells [34]. In the present study when CGRP was injected directly inside the TG, there was an increased mRNA expression of *Myd88* in TG after 6 h, whereas injecting Minocycline a glial inhibitor one hour before CGRP resulted in a decreased expression of *Myd88*. Results from our previous research showed that IG injection of CGRP lead to an increased gene expression of IL1-β and IL-6, glial activation and thermal hyperalgesia whereas Minocycline injected before CGRP reversed these results [13]. One inference which we can draw based on these results is that activating CGRP receptors may exert its proinflammatory/pronociceptive effect by triggering the NF-κB signaling pathway via *Myd88* adaptor protein downstream signaling involving *Irak1*, *Irak4*, and *Ikbkb*, *Ikbke*, and *Ikbkg* activity in the TG cells, however this needs to be further investigated. In a paclitaxel-induced peripheral neuropathy model, there was an increased in expression of Myd88 protein in CGRP positive neurons of DRG along with mechanical hyperalgesia [24]. When the animals were treated intrathecally with Myd88 inhibitor peptide (MIP), a reversal of mechanical hyperalgesia was observed [24]. Almost similar results were observed in a CCI model, where CCI resulted in mechanical and thermal hyperalgesia, with a concomitant increased in protein expression of Myd88 activity in CGRP and non-peptidergic isolectin B4 nociceptive neurons of DRG and astrocyte and microglial cells of spinal dorsal horn (SDH). There was a reversal of mechanical and thermal hyperalgesia when MIP was administered intrathecally, along with decreased Myd88 activity in DRG and SDH [25].

A highly upregulated gene after CGRP stimulation of glial cells was *Agt*, which is an NF-κB responsive gene. Agt is a vasoactive substance and it is produced locally in the central nervous system as shown by CSF/plasma ratio in an investigation [35]. The central nervous system (CNS) has a local renin–angiotensin system (RAS), and astrocytes synthesize angiotensinogen [36]. In a proteomic analysis of peripheral neuropathic pain patients, *Agt* was found to be significantly upregulated in cerebrospinal fluid of the patients and was found to have the highest discriminatory power between patients and healthy controls [37]. Candesartan, an angiotensin II type 1 receptor blocker was found to be effective in blunting the neuroinflammation by suppression of glial activation, stabilization of IκBα, nuclear translocation of NF-κB, imbalance in inflammatory cytokines, and Stat3 activation in both astroglial and microglial cells as well as in the rat model of neuroinflammation [38]. In our experimental condition, when glial cells were treated with Minocycline before CGRP stimulation, *Agt* was down regulated compared to only CGRP stimulated group (Table 1).

*Casp8* and *Eif2ak2*, gene which code for the protein kinase R (PKR), were significantly upregulated after CGRP stimulation. PKR has been reported to induce apoptosis by activation of the Fadd/Casp8/Casp3 and caspase 9 pathways [39,40] PKR is activated by proinflammatory cytokines, and in turn, activates inflammation-related pathways, including the pro-apoptotic JNK pathway and the proinflammatory NF-kB pathway [41,42]. Pharmacological inhibition of PKR has been shown to have neuroprotective effect by decreasing the neuronal loss and cleav Casp3 activity thus preventing neuroinflammation [43]. We observed that the expression of cleav Casp3 protein (active form), which is the executioner of apoptosis was mildly but significantly increased after IG CGRP injection. When Minocycline was injected before CGRP, there was a significant decrease in Casp3 expression in TG (Figure 4).

*Hmox1*, encodes for the enzyme heme oxygenase 1 (HO1), was highly upregulated when the glial cells were stimulated with CGRP and expression level didn’t change when glial cells were treated with Minocycline 30 min before CGRP stimulation. This enzyme has been reported to be highly expressed in the glial cells in brain neurodegenerative diseases, such as Alzheimer’s disease and Parkinson’s disease [44]. The HO 1 enzyme degrades heme into equimolar amounts of biliverdin, carbon monoxide (CO), and ferrous iron, thereby causing two opposing antioxidant and prooxidant functions. Biliverdin and CO act as antioxidants neuroprotective role against oxidative stress [45]. On the contrary, pathological iron deposition due to chronic activation of HO1 leads to irreversible neurological damage in several neurodegenerative diseases, including Alzheimer’s and Parkinson’s [44,46]. In PNS, the upregulation of HO1 was related to these opposing phenomena in Schwann cells in different conditions. The increased expression was associated with neuroprotection in culture, and with oxidative injury in damaged sciatic nerve due to increased iron metabolism in Schwann cells, thereby representing an important therapeutic agent/target for peripheral nerve degenerative diseases [47,48,49]. It has been reported that products derived from HO1 activity such as CO can inhibit NF-κB activity [50].

Certain genes were significantly and exclusively upregulated when glial cells were treated with Minocycline for 30 min before stimulation with CGRP compared to only CGRP stimulated group (Figure 2B). These genes were *Akt1*, *Egr1*, *Timp1*, *Ripk1*, and *Ltbr*. However, the mechanism through which minocycline exerts its anti-inflammatory and neuroprotective effect is not well-defined. However, minocycline exerts its activity possibly by inhibition of enzymes p38 MAPK, Casp1, Casp3, and iNOS, inhibition of glial activation [51,52,53,54].

Akt signaling pathway has been reported to improve glial energy metabolism and dependent neuron functioning, anti-inflammatory activity, and cell survival [55,56,57]. Minocycline has been reported to reverse the suppression of PI3-K/Akt pathway caused by glutamate and maintain the activation of this pathway by enhancing the phosphorylation of Akt triggered by PI3-K signaling, thereby preventing apoptosis of cerebellar granule neurons (CGN) [58]. Although in our cell culture experiment there was a significant upregulation of *Akt* when cells were treated with Min before CGRP stimulation, a similar effect was not observed when Min was injected before CGRP in TG (Figure 2B and Figure 3C).

The upregulation of matrix metalloproteinases (MMPs) has been linked to the development and maintenance of neuropathic pain. Timp-1 and Timp-2, the endogenous inhibitors of MMPs, are powerful agents for suppressing NP [59]. Minocycline has been reported to inhibit MMP2 and MMP9. Nerves from minocycline-treated diabetic rats showed an upregulation of *Timp1* mRNA [60]. We observed that Minocycline increased the expression of *Timp1* (Figure 2B). Minocycline also had an negative effect on the expression of Chemokine *Ccl2*, causing a downregulation in the expression of this chemokine (Table 1).

*Egr1*, which is a member of the family of immediate early genes, is widely used as a marker of neuronal activation and plasticity during memory formation [61]. Minocycline has been suggested to improve learning and memory through enhancing synaptic plasticity and synaptogenesis, which is suggested partly due to an increase in expression of *Egr1* [62]. In an in vivo experiment, when CGRP was injected in the TG, there was a significant decrease in expression of *Egr1* compared to control (Figure 3B). Whereas when Minocycline was injected one hour before CGRP the expression of *Egr1* was similar to control and significantly higher than the only CGRP group (Figure 3B). However, spinal *Egr1* is also reported to be involved in the maintenance of inflammatory pain. *Egr1* antisense treatment in rat resulted in deficits in the maintenance of mechanical allodynia [63] In *Egr1* knockout mice the acute nociception was unaltered, but hypersensitivity induced by formalin or complete Freund’s adjuvant was diminished [64]. An elevated level of spinal Egr1 protein correlated with the presence of pain and hyperalgesia and decrease in mRNA and protein expression after treatment corelated with the resolution of lameness and hyperalgesia. These results strengthen the role for Egr-1 in central neuronal plasticity underlying inflammatory or persistent pain [65].

Gene *Ltbr*, which is a cell surface receptor for lymphotoxin is a member of tumor necrosis factor receptor superfamily, showed an upregulation in Min + CGRP group (Figure 2B). It has been reported that Ltbr on glial cells contribute to demyelination and impede remyelination [66]. Activation of Ltbr signaling promotes astroglia and oligodendrocytic, but inhibits neuronal, lineage differentiation. Astroglial inactivation of NF-κB signaling compromises astroglial, but promotes neuronal, lineage differentiation [67]. There are no research reporting the connection between the upregulation of *Ltbr* and Minocycline’s anti-apoptotic, neuroprotective, or anti-inflammatory profile through glial inactivation or inhibition. The noteworthy fact is that several researchers have reported the deleterious effect of minocycline on neuronal survival in pre-clinical studies [68,69,70] and in a randomized phase III clinical trial involving amyotrophic lateral sclerosis (ALS) patients [71]. Disease progression in ALS patients given minocycline was faster than the control group and this effect, was not dose-dependent [71].

Though *Ripk1* was significantly upregulated in the Min + CGRP group (F = 1.99, *p* = 0.0417), it was slightly upregulated in CGRP stimulated group as well (F = 1.92, *p* = 0.0695) (Figure 2B and Table 1). Other genes which were upregulated even after Minocycline treatment and CGRP stimulation were *Eif2ak2*, *Hmox1*, *Irak1* and *4*, *Tnfrsf1a* and *Tradd*. The plausible explanation for this can be less than required dose or treatment duration of Minocycline for effective glial inhibition or overall, no or little effect of Minocycline on these genes. It seems that the Minocycline was not antagonizing the effect of CGRP. Rather, it induced the expression of genes which have anti-inflammatory and neuroprotective roles. However, all these factors need to be further investigated individually by exposing the glial cells to different concentration and for different time duration of Minocycline or treating glial cells with Minocycline alone.

CGRP causes peripheral and central sensitization through neuronal and glial activation in TG and spinal trigeminal nucleus [8], which contributes to the pathology of orofacial pain and migraine. We also observed that there was an increased mRNA expression of pro-inflammatory cytokines, neuronal and glial activation in TG [13], and cFos protein in second order neurons and GFAP immune reactivity in astroglia in trigeminal nucleus caudalis after CGRP administration in TG (Figure 5A,B). CGRP by acting through its receptor present on the neurons and glial cells can increase the expression of *iNOS* and stimulate nitric oxide (NO) release from glial cells [34,72]. An increase in *iNOS* expression and NO in rat dura mater stimulates the NF-κB pathway in resident meningeal macrophages [73], similar to the effect of CGRP on the glial cells as noted in the present study.

These findings raise a number of interesting queries requiring follow-up investigation to achieve the vision of mechanism-based pain diagnoses and treatments. To the best of authors’ knowledge this is the first study describing the effect of CGRP on NF-kB signal transduction on glial cells of trigeminal ganglion by upregulating the various genes of NF-kB pathway. Several genes of the IL-1 pathway (*Myd 88, Irak1, Irak4, Ikbkb, Ikbke, Ikbkg*) and some genes of the TNF pathway (*Tnfrsf1a, Tradd, Ripk*) were significantly upregulated along with the transcription factor *Rel*. Minocycline, which has gained popularity as putative glial inhibitor and was found to be effective in controlling CGRP induced thermal hyperalgesia in our previous study, resulted in the upregulation of certain genes which have anti-inflammatory properties (*Timp1, Akt1*), as well as neuronal activation marker (*Egr1*) and lead to negative regulation of inflammatory chemokine *Ccl2*. Collectively, the data demand further investigation concerning the role of glial cells in order to understand the immune responses in the trigeminal ganglion in orofacial pain.

## 4. Materials and Methods

### 4.1. Animals

The Animal Research Committee of the Tokushima University, Japan approved all animal care and experimental procedures (Protocol number—T27-78, date 4 November 2015; T30-75 date 27 September 2018). For all the experiments, 3–7 weeks old male *Sprague Dawley* rats were used. The rats were housed in groups of 2 or 3 rats per cage, under a controlled light cycle. Food and water were available ad libitum. All efforts were made to minimize animal suffering and to reduce the number of animals used.

### 4.2. Glial Rich Culture

Following the previously published work glial cells were dissociated from the TG by enzymatic treatment (0.125% collagenase P (Roche, Indianapolis, IN, USA), 0.02% DNase (Sigma-Aldrich, St. Louis, MO, USA) and 0.25% trypsin (Sigma, Kanagawa, Japan) as well as mechanical trituration [13,74]. The glial rich culture was prepared from the mixed culture at day 5, by detaching the cells by 5-min treatment with accutase (Nacalai Tesque, Inc., Kyoto, Japan). The cells were harvested from glial rich culture under control conditions (i.e., samples exposed to minimum essential medium, Life Technologies, Tokyo, Japan) or following overnight exposure (12 h) to 1 µM CGRP or cells treated with 20 nM Minocycline for 30 min followed by stimulation with 1 µM CGRP for 12 h.

### 4.3. NF-κB Signaling Pathway RT2 Profiler PCR Array

RNA was isolated from cells by direct lysis from the control and treatment conditions for mRNA arrays using RNeasy mini kit (Qiagen, Venlo, Netherlands, cat. Nos. 74104 and 74106) following manufacturer’s instructions. Each experimental condition was repeated in at least 3 independent experiments. Total 1 µg of isolated RNA was used to convert into cDNA using the RT2 First Strand Kit (Qiagen, Venlo, Netherlands, Cat. No. 53040) for all the samples. Genomic DNA elimination step was performed for all the samples. Resultant cDNA was used to perform real-time PCR using RT2 profiler PCR array (384-well (4 × 96) format) for rat NF-κB signaling pathway (Qiagen, Venlo, Netherlands, Cat.no. 330231 PARN-025ZA) in combination with RT2 SYBR Green qPCR Master mixes (Qiagen, Venlo, Netherlands, Cat. no. 330529) according to manufacturer’s instructions. Plates were read on Applied Biosystems 7900 HT qPCR machine using the following cycling condition: 1 cycle of 10 min at 95 °C followed by 45 cycles of 15 s at 95 °C and 1 min at 60 °C. SYBER Green fluorescence was monitored at the annealing step of each cycle. In this study, 96 genes were profiled on 9 samples with the PARN-025Z. The *C*_T_ cut-off was set to 35. Fold change/regulation using delta delta *C*_T_ method, (delta *C*_T_ (test group)-delta *C*_T_ (control group)). Fold Change is then calculated using 2^−∆∆*C*T^ formula. Genes which were included in this array analysis encodes for the proteins having role in various steps of NF-κB signaling pathway. These genes were NF-κB signaling ligands and receptors for example—*Tlr,* tumor necrosis factor receptor, lymphotoxin alpha, and others. Genes which encode for the proteins carrying out downstream signaling of NF-κB including various adapter proteins and kinases for example-*Myd88*, members of mitogen activated protein kinase family, *Irak*, receptor (TNFRSF)-interacting serine-threonine kinases. Genes which lead to the cytoplasmic sequestration of NF-κB and thereby enabling translocation into the nucleus include, for example, conserved helix-loop-helix ubiquitous kinase (*Chuk*), Ikbk, and others. The array also included various transcription factors like NF-κB light polypeptide gene enhancer in B-cells, *Rel*, *Egr1*, *Atf*, and others. NF-κB responsive genes causing an immune response, for example, chemokines (*Ccl2 and 5*), and apoptosis (B-cell leukemia/lymphoma 2 related protein) were also included in the array panel.

### 4.4. Intraganglionar Drug Administration

Either CGRP alone (10 µL of 10^−5^ M, (TOCRIS bioscience, Bristol, UK)), Minocycline (5 µL containing 10 µg Min, Nichi-Iko, Toyama, Japan) followed by CGRP with one-hour gap (Min + CGRP), or normal saline (10 µL) were injected in the TG according to the landmarks given in a previously published protocol [13,75]. The tissues were harvested 6 h after the injection for gene analysis and immunohistochemistry.

### 4.5. Quantitative Real-Time Polymerase Chain Reaction (qRT-PCR)

The qRT-PCR was done following our previously published work [13]. Briefly, total RNA was extracted from the trigeminal ganglion tissues and reverse-transcribed to cDNA. qRT-PCR was performed to evaluate the expression of *Akt, Myd88* and *Egr1*. The expression levels were normalized to an endogenous control, *Tbp* expression (*TATA box binding protein*). The primer sequences were selected from previously published research [76,77,78,79] (Table 2).

### 4.6. Immunohistochemistry

Immunohistochemical staining of TG and spinal trigeminal nucleus caudalis was done following our previously published work [13]. Briefly, the sections from TG were stained with primary antibody cleav Casp3 and secondary antibody (Table 3). The spinal trigeminal nucleus caudalis sections were stained with primary antibodies C-Fos and GFAP followed by secondary antibodies (Table 3). Nuclear staining was done using 4′,6-Diamidino-2-phenylindole dihydrochloride (DAPI, 1:100, Nacalai Tesque, Inc., Kyoto, Japan). Isotypes (goat IgG bs-0294P, and rabbit IgG bs-0295P, Bioss Antibodies, Boston, MA, USA) and only secondary antibodies were used as positive and negative control. Images were observed and acquired by confocal laser-scanning microscope (LSM 700, Carl Zeiss, Oberkochen, Germany).

### 4.7. Measurement of Staining Intensity for Cleav Casp3 Expression

To quantify the staining intensity of Casp3 in the trigeminal ganglia, the mean gray intensity of the regions of interest of TG containing several neuronal profiles were measured. A neuronal profile was considered as a neuron surrounded by associated satellite glial cells, which was identified by nuclear staining by DAPI. The relative staining intensity were determined using Fiji, Image J software (Ver 1.52, Wayne Rasband, National Institutes of Health, Bethesda, Maryland, USA). At first, the images acquired using confocal microscope were converted to RGB stack using image J (image > type > RGB stack), followed by adjusting the threshold to remove the background (image > adjust > threshold). Then, the mean grey value was measured. The fold change in staining intensity was defined as the mean change in relative intensity in the experimental condition (injected) when compared to mean levels of the non-injected control tissue, which was set equal to one [8].

### 4.8. Statistical Analysis

RT2 profiler PCR array data were analyzed using Gene Globe Data Analysis Center by Qiagen. The *p* values were calculated using Student’s *t*-test of the replicate 2^−∆*C*T^ values for each gene in the control group and treatment groups. q RT-PCR and immunohistochemistry data were analyzed using SPSS software version 25 (IBM, New York, NY, USA). A *p*-value less than 0.05 was considered statistically significant.

## Figures and Tables

**Table 1:** Gene regulation in the glial cells of Trigeminal ganglion of NF-κB signaling pathway using RT PCR array when glial cells were stimulated with CGRP compared to no treatment control group, when glial cells were treated with Minocycline for 30 min followed by CGRP stimulation (Min + CGRP) compared to control and CGRP and Min + CGRP group. (Data is arranged in the increasing order of *p*-value of CGRP vs. control group).

Gene Symbol	CGRP vs. Control		Min + CGRP vs. Control		CGRP vs. Min + CGRP	
	Fold Regulation	*p*-Value	Fold Regulation	*p*-Value	Fold Regulation	*p*-Value
Angiotensinogen (Agt)	4.53	0.00507	1.68	0.3879	2.7	0.028231
Heme oxygenase (decycling) 1 (Hmox1)	3.23	0.00524	3.65	0.00207	−1.13	0.353707
Inhibitor of kappa light polypeptide gene enhancer in B-cells, kinase epsilon (Ikbke)	2.08	0.00939	1.42	0.13566	1.47	0.032139
Inhibitor of kappa light polypeptide gene enhancer in B-cells, kinase beta (Ikbkb)	1.75	0.01188	1.45	0.10942	1.21	0.303584
PC4 and SFRS1 interacting protein 1 (Psip1)	2.14	0.01558	1.46	0.13784	1.47	0.010853
V-rel avian reticuloendotheliosis viral oncogene homolog (Rel)	1.81	0.01719	1.23	0.35578	1.47	0.007833
Interleukin-1 receptor-associated kinase 4 (Irak4)	1.88	0.01835	1.8	0.04689	1.05	0.842815
Tumor necrosis factor receptor superfamily, member 1a (Tnfrsf1a)	1.9	0.02122	1.7	0.0305	1.12	0.419134
TNFRSF1A-associated via death domain (Tradd)	1.73	0.02449	1.69	0.02557	1.03	0.841517
Interleukin-1 receptor-associated kinase 1 (Irak1)	1.8	0.02899	1.76	0.01546	1.02	0.806749
Eukaryotic translation initiation factor 2-alpha kinase 2 (Eif2ak2)	2.22	0.03561	1.6	0.04048	1.39	0.079264
(Myeloid differentiation primary response gene 88) Myd88	1.77	0.03856	1.36	0.21353	1.3	0.080385
Inhibitor of kappa light polypeptide gene enhancer in B-cells, kinase gamma (Ikbkg)	1.6	0.04495	1.41	0.13474	1.13	0.599914
Caspase 8 (Casp8)	1.73	0.04646	1.81	0.06508	−1.04	0.721599
V-rel reticuloendotheliosis viral oncogene homolog A (avian) (Rela)	1.94	0.05081	1.63	0.18091	1.19	0.470757
Epidermal growth factor receptor (Egfr)	2.77	0.05516	2.06	0.18127	1.35	0.336574
V-raf-leukemia viral oncogene 1 (Raf1)	1.79	0.05592	1.67	0.10164	1.08	0.732804
TIMP metallopeptidase inhibitor 1 (Timp1)	1.5	0.05971	1.69	0.00688	−1.12	0.266271
CREB binding protein (Crebbp)	1.76	0.06267	1.8	0.12331	−1.02	0.782512
Receptor (TNFRSF)-interacting serine-threonine kinase 1 (Ripk1)	1.92	0.06956	1.99	0.04171	−1.03	0.84311
Tnf receptor-associated factor 6 (Traf6)	1.52	0.07076	1.57	0.06166	−1.03	0.772281
Jun oncogene (Jun)	1.66	0.09329	1.98	0.10892	−1.2	0.407036
Mitogen activated protein kinase 3 (Mapk3)	1.53	0.09343	1.47	0.07564	1.04	0.771937
Toll-like receptor 4 (Tlr4)	1.77	0.09811	1.42	0.36215	1.25	0.53273
Interleukin 1 receptor, type I (Il1r1)	2.07	0.1041	1.58	0.3264	1.31	0.200475
Activating transcription factor 1 (Atf1)	1.87	0.10581	2.07	0.05969	−1.11	0.50731
Tnf receptor-associated factor 3 (Traf3)	1.61	0.11009	1.26	0.43188	1.27	0.063863
Tumor necrosis factor receptor superfamily, member 1b (Tnfrsf1b)	1.99	0.11131	1.57	0.27579	1.27	0.062087
Fas (TNFRSF6)-associated via death domain (Fadd)	1.45	0.12247	1.07	0.771	1.35	0.178473
Mitogen activated protein kinase kinase kinase 1 (Map3k1)	1.63	0.12685	1.33	0.36626	1.23	0.198982
Signal transducer and activator of transcription 1 (Stat1)	1.57	0.13515	1.19	0.4605	1.32	0.189901
Mitogen activated protein kinase kinase 3 (Map2k3)	1.53	0.1407	1.43	0.12152	1.07	0.760551
Toll-like receptor 3 (Tlr3)	2.61	0.14249	1.77	0.49273	1.48	0.021363
Nuclear factor of kappa light polypeptide gene enhancer in B-cells 1 (Nfkb1)	1.62	0.1512	1.51	0.18631	1.07	0.608812
Colony stimulating factor 1 (macrophage) (Csf1)	1.6	0.15174	1.15	0.7453	1.39	0.034653
Toll interacting protein (Tollip)	2.42	0.17346	1.89	0.1088	1.28	0.408384
Lymphotoxin alpha (TNF superfamily, member 1) (Ltbr)	1.48	0.17467	2.39	0.00038	−1.62	0.042208
CASP8 and FADD-like apoptosis regulator (Cflar)	1.6	0.17536	1.33	0.40881	1.2	0.183055
Tumor necrosis factor receptor superfamily, member 10b (Tnfrsf10b)	1.6	0.18011	2.1	0.08054	−1.31	0.235267
Conserved helix-loop-helix ubiquitous kinase (Chuk)	1.56	0.18368	1.55	0.17499	1.01	0.970965
B-cell CLL/lymphoma 3 (Bcl3)	3.25	0.19355	2.21	0.50387	1.47	0.062677
FBJ osteosarcoma oncogene (Fos)	2.24	0.19478	2.69	0.06886	−1.2	0.459281
Interferon regulatory factor 1 (Irf1)	1.83	0.19915	1.34	0.59688	1.37	0.085414
SMAD family member 4 (Smad4)	1.71	0.20071	1.69	0.1561	1.01	0.952726
TANK-binding kinase 1 (Tbk1)	1.71	0.20588	1.28	0.51904	1.33	0.146051
Receptor-interacting serine-threonine kinase 2 (Ripk2)	1.7	0.20951	1.81	0.13488	−1.07	0.604089
P300/CBP-associated factor (Kat2b)	1.66	0.22458	1.54	0.21379	1.08	0.519346
Tnf receptor-associated factor 2 (Traf2)	1.32	0.23271	1.31	0.19467	1	0.993216
V-akt murine thymoma viral oncogene homolog 1 (Akt1)	1.23	0.24355	1.94	0.03025	−1.57	0.071343
Nuclear factor of kappa light polypeptide gene enhancer in B-cells inhibitor, alpha (Nfkbia)	2.44	0.27856	1.4	0.98802	1.74	0.002189
Intercellular adhesion molecule 1 (Icam1)	1.57	0.30001	1.44	0.38205	1.08	0.716784
Activating transcription factor 2 (Atf2)	2.05	0.32496	1.87	0.36302	1.1	0.525649
Toll-like receptor 6 (Tlr6)	1.36	0.33783	1.11	0.79499	1.23	0.301466
CD40 molecule, TNF receptor superfamily member 5 (Cd40)	4.09	0.34993	2.55	0.95769	1.61	0.060072
Fas ligand (TNF superfamily, member 6) (Faslg)	−8.17	0.37086	−4	0.28782	−2.04	0.346818
Interleukin 1 alpha (Il1α)	−7.98	0.37275	−15.36	0.28247	1.93	0.223631
Similar to Tnf receptor-associated factor 1 (LOC687813)	1.75	0.40218	1.56	0.50132	1.13	0.594782
Interleukin-1 receptor-associated kinase 2 (Irak2)	1.61	0.40882	1.18	0.90517	1.37	0.2152
Toll-like receptor 9 (Tlr9)	−1.27	0.48708	−1.9	0.14612	1.5	0.296125
B-cell CLL/lymphoma 10 (Bcl10)	1.76	0.49896	2.05	0.21512	−1.16	0.352739
Caspase recruitment domain family, member 10 (Card10)	1.37	0.59587	2.02	0.05805	−1.47	0.015496
Bcl2-like 1 (Bcl2l1)	1.46	0.61736	1.96	0.14253	−1.34	0.059029
Toll-like receptor 2 (Tlr2)	1.71	0.64305	1.25	0.87114	1.37	0.24527
Early growth response 1 (Egr1)	1.65	0.6683	4.17	0.00864	−2.53	0.007471
Coagulation factor II (thrombin) receptor (F2r)	1.15	0.70587	−1.21	0.62531	1.39	0.324815
Tumor necrosis factor (TNF superfamily, member 2) (Tnf)	1.1	0.72791	1.37	0.85781	−1.24	0.518336
B-cell leukemia/lymphoma 2 related protein A1d (Bcl2a1)	1.53	0.74497	1.15	0.49908	1.33	0.446459
Caspase 1 (Casp1)	1.72	0.75883	1.31	0.96179	1.31	0.469404
Nuclear factor of kappa light polypeptide gene enhancer in B-cells 2, p49/p100 (Nfkb2)	1.28	0.76734	1.74	0.29266	−1.36	0.323335
Interleukin 1 beta (Il1β)	2.81	0.86644	1.55	0.43662	1.82	0.288445
Chemokine (C-C motif) ligand 2 (Ccl2)	2.42	0.92611	−1.07	0.43466	2.59	0.011511
Toll-like receptor 1 (Tlr1)	1.45	0.94286	1.09	0.63331	1.33	0.266362
Baculoviral IAP repeat-containing 3 (Birc3)	1.56	0.99554	1.52	0.98963	1.03	0.983089

**Table 2:** Primers used for quantitative RT-qPCR.

ID	Sequence (5’-3’)
*Akt F*	CTTTATTGGCTACAAGGAACGG
*Akt R*	CAGTCTGAATGGCGGTGGT
*Egr1 F*	CCAGTGCCCACCTCTTACTC
*Egr1 R*	TGCAGACTGGAAGGTGCTG
*Myd88 F*	TTCTCCAACGCTGTCCTGTC
*Myd88 R*	AACTGAGATGTGTGCCCAGG
*Tbp F*	TGGGATTGTACCACAGCTCCA
*Tbp R*	CTCATGATGACTGCAGCAAACC

**Table 3:** List of antibodies used in immunohistochemistry.

Antibody Name	Company	Dilution
Cleaved Caspase-3	Cell-signalling technology (9664)	1:800
C-Fos	Enzolife (ALX-210-130-C100)	1:20
GFAP	Abcam (ab53554)	1:500
Alexa Fluor 488	Abcam (ab150129)	1:200
Alexa Fluor 555	Abcam (ab150074)	1:200
Alexa Fluor 488	Thermo Fisher Scientific (A11008)	1:200
